# Cancer-related emergency and urgent care: expanding the research agenda

**DOI:** 10.1186/s44201-022-00005-6

**Published:** 2022-06-14

**Authors:** Nonniekaye Shelburne, Naoko Ishibe Simonds, Roxanne E. Jensen, Jeremy Brown

**Affiliations:** 1 Division of Cancer Control and Population Sciences, National Cancer Institute, Rockville, MD 20850 USA; 2 The Scientific Consulting Group, Inc., Gaithersburg, USA; 3 Office of Emergency Care Research, National Institute of Neurological Disorders and Stroke, Bethesda, USA

**Keywords:** Emergency care, Emergency medicine, Oncology, Cancer, Cancer care delivery, Urgent care

## Abstract

**Purpose of review:**

Cancer-related emergency department (ED) visits often result in higher hospital admission rates than non-cancer visits. It has been estimated many of these costly hospital admissions can be prevented, yet urgent care clinics and EDs lack cancer-specific care resources to support the needs of this complex population. Implementing effective approaches across different care settings and populations to minimize ED and urgent care visits improves oncologic complication management, and coordinating follow-up care will be particularly important as the population of cancer patients and survivors continues to increase. The National Cancer Institute (NCI) and the Office of Emergency Care (OECR) convened a workshop in December 2021, “Cancer-related Emergency and Urgent Care: Prevention, Management, and Care Coordination” to highlight progress, knowledge gaps, and research opportunities. This report describes the current landscape of cancer-related urgent and emergency care and includes research recommendations from workshop participants to decrease the risk of oncologic complications, improve their management, and enhance coordination of care.

**Recent findings:**

Since 2014, NCI and OECR have collaborated to support research in cancer-related emergency care. Workshop participants recommended a number of promising research opportunities, as well as key considerations for designing and conducting research in this area. Opportunities included better characterizing unscheduled care services, identifying those at higher risk for such care, developing care delivery models to minimize unplanned events and enhance their care, recognizing cancer prevention and screening opportunities in the ED, improving management of specific cancer-related presentations, and conducting goals of care conversations.

**Summary:**

Significant progress has been made over the past 7 years with the creation of the Comprehensive Oncologic Emergency Research Network, broad involvement of the emergency medicine and oncology communities, establishing a proof-of-concept observational study, and NCI and OECR’s efforts to support this area of research. However, critical gaps remain.

## Introduction

An estimated 1.9 million new cancer diagnoses were made in the United States (US) during 2021 [1], with increasing frequency of treatment provided in the outpatient setting. Managing unexpected or acute side effects and adverse events from cancer and cancer therapy poses a challenge to ambulatory care patients, their caregivers, and the healthcare system. This often results in patients and caregivers seeking care outside of their oncology team. Retrospective studies of US emergency department (ED) visits by patients presenting with a cancer-related complaint report 4.2 million adult and nearly 300,000 pediatric visits annually [2, 3].

Due to varying levels of oncology care model implementation, resource availability, and the complexity of cancer treatment, patients experiencing acute complications (e.g., fever, shortness of breath) rely on urgent and emergency care providers to efficiently triage and stabilize medical presentations and coordinate follow-up care with oncology care teams. Clinicians and researchers continue to develop and test assessment tools that identify patients at greater risk for high care needs and strategies to provide support that minimizes the need for urgent and emergency care. Initiatives, such as the Centers for Medicare & Medicaid Services Oncology Care Model, encourage innovation to improve quality of care and reduce Medicare spending through financial incentives [4]. The results of these efforts have been mixed. Some of the barriers to providing patient-centered care for unplanned care needs include the limited ability of non-oncology providers to access cancer patient treatment documentation, a lack of evidence-based cancer-specific triage and management pathways, and fractured provider-to-provider communication.

In 2014, the National Cancer Institute (NCI) and the Office of Emergency Care Research (OECR) began collaborating to address the paucity of evidence surrounding cancer-related ED visits. They held the first scientific meeting on the topic in 2015 and published a research agenda to advance the understanding of emergency care of cancer patients. The identified knowledge gaps covered characterization of the cancer population utilizing ED care, the management of the patient with febrile neutropenia and acute events, and the role of palliative care in the ED [5].

To update the state of knowledge, evidence gaps, and research recommendations, the NCI and OECR convened a virtual, public workshop in December 2021, “Cancer-related Emergency and Urgent Care: Prevention, Management, and Care Coordination” [6]. The workshop brought together over 100 participants with clinical and research expertise in oncology, urgent care, emergency medicine, healthcare delivery, nursing, social work, and patient advocacy. The goal was to reassess the knowledge gaps and research recommendations in cancer-related urgent and emergency care prevention, management, and coordination of care. This report provides an overview of the current landscape of oncologic urgent and emergency care and the research recommendations identified by workshop participants.

## Progress and advances in cancer-related urgent and emergency care

Since the 2015 Workshop identified research opportunities and established new collaborations across oncology and emergency medicine, progress has been made in understanding the care needs of those experiencing oncologic complications in the urgent and emergency care setting. NCI has a stated research interest in cancer and emergency medicine that focuses on the following: (1) utilization and drivers of cancer-related emergency care, (2) risk stratification, prediction models, and intervention strategies, and (3) minimizing emergency care use [7]. Numerous funding opportunity announcements are available to support new grant submissions across these interest areas (Table 1). Since 2015, NCI has seen a rise in grant application submissions related to oncologic complications. For example, recently funded applications include characterization of patients diagnosed with cancer in the ED, implementation of an ED cervical cancer screening program, and development of an ED-risk stratification tool for immune-related adverse events. In addition, NCI funds projects assessing and managing cancer-related symptoms and toxicities in the oncology setting that include ED visits, a key healthcare utilization study endpoint.

**Table 1:** NIH notice of special interest and funding opportunity announcements: cancer-related urgent and emergency care

Grant mechanism	NOSI/FOA activity code	NOSI/FOA title	Expiration date ***Unless reissued***
Multiple	NOT-NS-20-005	Research in the Emergency Setting	September 8, 2022
R01	PAR-21-190	Modular R01s in Cancer Control and Population Sciences (R01 Clinical Trial optional)	March 8, 2024
R01	PA-20-185	NIH Research Project Parent Grant (Parent R01 Clinical Trials not allowed)	May 8, 2023
R01	PAR-21-035	Cancer Prevention and Control Clinical Trials Grant Program (R01 Clinical Trial required)	January 8, 2024
R01	PA-20-183	NIH Research Project Grant (Parent R01 Clinical Trial Required) — *NCI clinical trial applications must use PAR-21-035*	May 8, 2023
R03	PAR-20-052	NCI Small Grants Program for Cancer Research (NCI Omnibus R03 Clinical Trial optional)	January 8, 2023
R21	PAR-21-341	Exploratory Grants in Cancer Control (R21 Clinical Trial optional)	October 9, 2024
P01	PAR-20-077	NCI Program Project Applications (P01 Clinical Trial optional)	May 8, 2023

*NIH* National Institutes of Health, *NOSI* Notice of Special Interest, *FOA* Funding opportunity announcement, *PAR* Program announcement, *NCI* National Cancer Institute

Another outcome of the 2015 Workshop was the establishment of the Comprehensive Oncologic Emergency Research Network (CONCERN), created to accelerate knowledge generation, synthesis, and translation of oncologic emergency medicine research [8]. This research network conducted a prospective, observational study of adult patients with active cancer presenting to the ED with oncologic complaints. CONCERN study publications include a descriptive study on key population characteristics [9], a validation of the Emergency Severity Index triage tool during active cancer treatment [10], the characterization of pain severity, medication utilization, and clinical outcomes [11], and a descriptive study on observation unit care utilization [12].

Even with this tangible progress, the 2021 Workshop participants noted that many challenges remain. The themes that emerged from the current workshop were (1) utilization and prediction, (2) care delivery models and strategies, (3) cancer prevention and screening, (4) managing acute cancer-related presentations, (5) cancer-related goals of care conversations, and (6) designing and conducting cancer-related urgent and emergency care research studies (Table 2).

**Table 2:** 2021 workshop recommendations: research opportunities in cancer-related urgent and emergency care

**ED utilization and prediction**	
1. Establish standard definitions and measures of preventable or avoidable oncology-related urgent care and ED visits.	
2. Establish consistent methodologies to study ED utilization for cancer care.	
3. Characterize individual, system, and societal drivers of unscheduled cancer-related medical care (e.g., reasons for visit, rural versus urban settings, socioeconomic).	
4. Characterize individual, system, and societal drivers of new cancer diagnoses identified in the ED setting.	
5. Develop predictive models to identify those at high risk for unscheduled cancer care based on manageable or modifiable factors.	
6. Create data linkages among existing sources to address utilization and risk prediction knowledge gaps (e.g., EHR, claims/administrative data, PRO).	
7. Utilize technologies, such as natural language processing, to enhance capture of meaningful and actionable data from registry and EHR that are relevant to managing unscheduled cancer care visits.	
8. Conduct large, prospective observational studies with detailed information on patient symptoms, cancer history, treatment history, unscheduled care management interventions, outcomes, and disposition to characterize utilization of urgent and emergent care across care settings.	
**Care delivery models and strategies**	
9. Identify and test existing and novel oncology and emergency medicine healthcare models to evaluate their impact on unscheduled cancer care prevention, management, care coordination, and patient outcomes, including effectiveness, cost, and patient acceptability.	
10. Characterize pre-, peri-, and post-pandemic urgent and emergent care use, prevention and management strategies, and outcomes for cancer-related needs.	
11. Develop and test modifiable risk factor reduction interventions such as telemedicine, remote home monitoring, and PRO measures across various settings and populations.	
12. Test and validate the use of artificial intelligence and machine learning for cancer-related symptom monitoring and management in the outpatient oncology setting to identify and intervene before they become severe or uncontrolled.	
13. Employ standard measures to better characterize outcome and cost of oncology, urgent, and emergency care delivery models.	
14. Characterize the impact of specialty knowledge and resources that improve cancer outcomes in urgent and emergency care settings (e.g., oncology providers in the ED).	
**Cancer prevention and screening in ED**	
15. Characterize the population who is relying on the ED for planned care and may most benefit from cancer prevention and screening services.	
16. Develop and test strategies to increase cancer screening uptake in the ED while accounting for competing resources.	
17. Develop, test, and implement information technology solutions to identify ED patients that should be offered cancer screening and follow-up.	
18. Identify the barriers to cancer screening and prevention referrals and follow-up post ED visit and test strategies to improve outcomes, considering different resource and care settings.	
**Managing cancer-related presentations in urgent care and the ED**	
*Risk stratification in ED*	
19. Develop and validate new ED-oncology-specific risk stratification tools for severity of presentation, resource utilization, and disposition (e.g., for neutropenic fever, pulmonary embolism, immune-related adverse events).	
20. Develop, test, and adopt clinically viable and sustainable biomarkers and rapid diagnostics to risk stratify patients presenting with oncologic emergencies.	
21. Develop effective quick assessment tools using EHR data for cancer patient triage and care delivery in urgent and emergent care settings.	
*Clinical pathways*	
22. Integrate existing and new cancer symptom and adverse event management evidence into emergency medicine accelerated pathways and test effectiveness and implementation across settings and populations.	
23. Determine which care pathways are generalizable and scalable across care settings and populations.	
24. Create efficient communication pathways for smaller ED to access resource rich care systems.	
25. Study and compare costs and efficiencies across care settings, including behavioral economics, identification of CPT codes that will be/will not be reimbursed, duplication of imaging studies, laboratory tests, etc.	
*Disposition*	
26. Identify individual, system, and societal factors associated with hospitalization after a cancer-related ED visit.	
27. Develop and test strategies to improve post-urgent and emergency follow-up care and communication.	
28. Examine methods to improve interoperability of medical record sharing between specialty care settings (e.g., oncology, emergency medicine) and institutions (e.g., academic, community) to support decision making and follow-up care for unscheduled cancer care.	
**Cancer-related goals of care in the ED**	
29. Characterize the unmet palliative care or end-of-life needs of cancer patients utilizing emergency care services.	
30. Develop, test, and adopt symptom management assessment tools to identify patients with cancer that may benefit from palliative care referrals for management of symptoms, care support, and/or goals of care conversations (e.g., PCaRES — facilitated assessment of eligibility for palliative care when in the ED)	
31. Develop, test, and implement interventions to communicate and meet individual goals of care and minimize urgent and emergent care needs (e.g., offer hospice services, palliative care services).	
32. Integrate palliative care into clinical trials in advanced cancer patients.	
33. Test various care coordination and navigation strategies post-urgent care or ED visit to identify barriers and facilitators to improve outcomes.	
**Designing and conducting oncologic emergency research studies**	
34. Leverage prior ED research methods for other diseases/symptoms (e.g., cardiovascular events) to develop, test, and implement oncology specific care pathways.	
35. Engage urgent care, emergency medicine, oncology, primary care, clinical care team members, informatics, patients, advocacy groups, community partners, and other key stakeholders in cancer-related urgent and emergency care study design to enhance the translation of study findings and improve application to diverse populations and settings.	
36. Assess existing oncologic emergency research fellowships to identify facilitators to success and identify opportunities to expand mentor opportunities across programs.	
37. Promote more standardized EHR data collection for oncology patient encounters in the emergency care setting.	
38. Develop standard cancer ED visit data collection elements for observation and intervention studies to promote consistent assessment parameters and outcome measurement.	
39. Identify, assess, and integrate evolving evidence into practice guidelines with an initial focus on high-risk and high-frequency symptom presentations, considering the resource variability of urgent and emergent care settings.	

*ED* Emergency department, *PRO* Patient-reported outcome, *EHR* Electronic health record, *CPT* Current procedural terminology, *PCaRES* Palliative care screening

## Workshop findings and recommendations

### ED utilization and prediction

Four percent of adult ED visits in the USA are for cancer-related reasons. These ED visits occur across the cancer care continuum, including initial diagnosis, active treatment, survivorship, and at end of life [3, 13]. Higher ED use has been reported for some cancers (e.g., lung, brain, leukemia) compared to others (e.g., prostate, breast) especially during the first year after diagnosis [14–16], and frequent reasons cited for these visits are symptom management (e.g., pain, fever or infection, respiratory distress, gastrointestinal issues). Risk factors associated with ED visits include chronic conditions, frailty, advanced-stage disease, being at sociodemographic risk, and prior ED use [9, 14–18]. Significantly, cancer-related ED visits result in an inpatient admission at a rate 3.5 times than that of non-cancer-related ED visits [3, 9, 13, 17]. Despite growing knowledge of cancer-related ED utilization and population characteristics, workshop panelists noted that there are continued gaps in the epidemiology literature, some of which are limited by the quality of available data and inconsistent definitions of avoidable or preventable ED visits.

ED visits and unplanned hospitalization are common in the cancer population, and better prediction tools are needed to identify patients at increased risk for high symptom burden, adverse events, or poor outcomes. By identifying high-risk patients for whom proactive care management might be warranted, strategies such as increased symptom assessments (e.g., electronic symptom monitoring, virtual follow-up) and addressing social needs (e.g., prescription plans for symptom control medications, transportation support) could help prevent some unscheduled care. For example, machine learning-based risk tools for mortality, ED visit, and hospital admission outcomes have been developed and validated for pre-treatment [19] and after starting treatment [20]. Although these machine learning and artificial intelligence models have the potential to predict outcomes, additional studies addressing limitation of single-center studies, testing in single electronic health records (EHR) type, and population representation are needed. Additionally, there is a growing body of literature supporting the use of patient-reported outcomes to facilitate cancer symptom assessment [21, 22] and its impact on daily activities and quality of life. However, these assessments have not focused on the risk of unplanned care. Additional studies on which patient-level variables (e.g., clinical risk factors, prior ED use, and sociodemographic factors) should be included in these models, and their impact on healthcare utilization is needed.

To better address the needs of cancer patients, understanding their experiences in the urgent and emergency care setting is required. Thus far, patient perspectives “in the setting of an unplanned acute care event remain poorly described” [23]. Some cancer survivor workshop participants shared their experiences managing oncologic complications, reporting challenges in navigating the healthcare system, such as not receiving clear instructions on how to contact the oncology team for guidance with acute events. When they did turn to the ED for care, they frequently felt they had to advocate for themselves, and that ED providers did not always take their concerns seriously. One panelist suggested that quicker time to triage and provider assessment are warranted for patients with cancer, in a model similar to that provided in pediatric emergency departments. Taken together, research is needed to better understand how care coordination and resources can be improved for patients.

Workshop discussions also identified several research opportunities to better understand urgent and ED utilization and improve risk prediction of visits. A first step is to consistently define what is an avoidable or preventable urgent care or ED visit; standardizing this definition is essential for outcome measures in future research in utilization and prediction. Linking existing data sources, such as EHR and claims and registry data, and applying machine learning algorithms could identify cancer patients at risk of unscheduled cancer-related needs, as well as help characterize the individual, system, and societal drivers of acute care. Furthermore, additional prospective observational studies of cancer-related ED visits, including underrepresented populations and settings, are warranted.

## Care delivery models and strategies

Cancer-related ED visits result in hospital admission at rates well above that of non-cancer patients (60% vs. 16%) [3]. More significantly, it has been estimated that 30 to 60% of these admissions can be prevented [24, 25], and nearly 50% of oncology care cost results from preventable toxicities [26, 27]. Implementing effective care delivery models across different care settings and populations to minimize urgent care and ED visits will be particularly important as the population of cancer patients and survivors continues to increase.

Workshop speakers presented several care delivery strategies that have been tested to minimize unscheduled ED visits and inpatient admissions as well as improve care in these patients. For example, access and care coordination, such as patient navigation [28], web-based symptom self-reporting [29], oncology urgent care clinics, nurse triage [30], and post-discharge interventions [31] have all been shown to reduce ED visits and/or hospitalization. Some ED and urgent care center observation units provide outpatient acute care that lasts more than 24 h in duration but is typically less than 48 h, and these have been reported to reduce inpatient admissions [32]. They are, however, an underused and understudied resource. The recent global SARS-CoV-2 pandemic also required hospitals and outpatient practices to develop and implement cancer-related symptom management strategies focused on preventing ED visits to reduce the demands on the healthcare system as well as to minimize exposure, and the prevalence of telemedicine has grown rapidly. More studies investigating the impact, cost, and sustainability of these strategies on cancer outcomes are needed.

While these care delivery models have been recognized as being important to improving the care of cancer patients seeking unscheduled care, there is no clear “best practice” model. Furthermore, it is not obvious how to scale up these resource-intensive models, and participants noted that some were cumbersome to implement. Participants noted that less than half of cancer patients call their care team for advice prior to seeking unscheduled care, even though many oncology teams provide a 24-h phone consultation service. Clearly, there is a critical need to better align the needs of cancer patients and caregivers to not only minimize unscheduled care needs but also provide supportive and accessible care. During the workshop, several important next steps were identified, including developing and testing care delivery models and strategies that have the most impact on minimizing urgent care and ED use, improving patient outcomes (e.g., mortality, hospital length o stay), and lowering costs of care delivery.

## Cancer prevention and screening in the ED

Approximately, 2 million adults report using the ED as their usual source of care [33] presenting the opportunity to provide cancer control information and care. Those who seek care in the ED are more likely to be younger, socioeconomically disadvantaged, and racial/ethnic minorities [33] who do not have access to preventive care. There is a growing body of evidence showing that providing care that is typically thought of as preventive (e.g., vaccinations, tobacco, and alcohol cessation) while in the ED to be effective [34, 35]. Moreover, it was noted that the ED is a “target-rich” environment with many patients needing cancer screening (e.g., Pap smear, mammography, colonoscopy), especially among those who do not have ready access to primary care. Participants further noted that using the wait time in the ED could be an opportunity to educate patients as they tend to be receptive. However, there were questions as to (1) how to incorporate screening and prevention efforts without overburdening staff, (2) which screening tests can and should be performed in the ED, and (3) how to ensure appropriate follow-up care.

To address these questions, a few key steps were identified by workshop participants, starting by characterizing the population who rely on the ED for planned care and who may most benefit from cancer prevention and screening services. Next, strategies to increase cancer screening uptake in the ED should be developed and tested. These could include providing simple or enhanced (e.g., mHealth) referral to local screening resources, placing a cancer screening/prevention unit near the ED, or actual in-ED screen. The development of technology solutions (e.g., smart EHR) to identify ED patients appropriate for cancer prevention and screening was highlighted as another research opportunity. Finally, identifying the best approach to ensure appropriate follow-up, such as using patient navigators, to improve preventive healthcare delivery to ED patients should be explored.

## Managing cancer-related presentations in urgent care and the ED

Clinical pathways support evidence-based risk stratification and clinical decision-making for rapid assessment and treatment in the ED with the goal to predict resource utilization and improve patient outcomes. Pathways that are adopted address common clinical presentations and are easy to use. Pathways in non-cancer populations have been widely adopted in the ED, such as the HEART score for myocardial infarction with undifferentiated chest pain, pulmonary embolism rule-out criteria (PERC) for pulmonary embolism, and coronavirus rule-out criteria (CORC) for COVID-19. Workshop participants expressed that existing cancer-focused pathways, such as the Multinational Association for Supportive Cancer Care (MASCC) and Clinical Index of Stable Febrile Neutropenia (CISNE) for febrile neutropenia in cancer patients, are cumbersome and have not been validated prospectively. Furthermore, it was noted that frequently, the tools that appear promising at a single site are less effective when tested in other care settings.

Throughout the workshop, participants stated the need to develop and validate ED oncology-specific clinical pathways for commonly observed oncologic complications. Workshop attendees noted the need to adapt, test, and integrate existing care pathways used in oncology care settings into the ED and conduct research where gaps remain. Specifically, tools to assist with differential diagnosis of febrile neutropenia, pulmonary embolism, and immune-related adverse events for severity, resource utilization, and disposition were identified as an urgent need. In addition, it is important to develop and test diagnostic and prognostic biomarkers that can risk stratify patients, which in the ED setting, should have the following key features: easy to measure, fast time to result, and excellent predictive value. Comparing costs and efficiencies across care settings will support care pathways dissemination and implementation. Given the vital role these tools play in the ED, deriving risk stratification and clinical decision-making tools in a multicenter study that is subsequently validated in other populations and care settings is critical.

A lack of cancer-specific pathways in the ED also contributes to the high hospital admission rate for cancer-related ED visits. Tools that assess individual, system, and societal factors to inform the management of febrile neutropenia, for example, could assist providers in determining the safety of discharging patients home on oral antibiotics instead of admitting them for intravenous antibiotics. The goal of discharge to home comes with the need for improved post-ED communication between patients and provider teams. Workshop participants noted the need to standardize EHR data collection for oncology patient encounters in the emergency care setting to allow for sharing of medical records and supporting timely follow-up.

## Cancer-related goals of care in the ED

Understanding the complexity of acute care ED visits for advanced stage patients with cancer and aligning care based on their needs and desires are an important research topics. Although there has been research demonstrating that specialized palliative and hospice care improves quality-of-life in cancer patients, palliative and hospice care referral is highly variable [36, 37], in both oncology practice and the ED. However, many questions remain as to what approach and services best address these patients’ and caregivers’ needs. These include deciding which provider leads the “goals of care” conversation (social worker, nurse, physician), when the timing of the intervention should occur (episode of care: pre-, within-, post-ED visit), how it should be delivered (face to face, telephone), and what type of care should be provided (specialty palliative care, hospice care, community paramedicine, caregiver support).

This is further complicated by inadequate communication of care preferences throughout the various stages of disease progression. The “goals of care” conversation should preferentially occur in the outpatient oncology setting, yet it has been estimated that only about a third of patients with advanced cancer have this conversation with their oncologists [38]. When patients with advanced or late-stage cancer arrive in the ED seeking care, goals of care conversations can become a crisis communication, potentially leading to undesired invasive interventions. Although the ED visit is an opportunity to introduce such conversations, emergency physicians are often unsure of their roles in initiating these conversations and lack a practical method to guide discussions with seriously ill patients about their values and preferences in a time-pressured environment [39].

Several important research opportunities were identified to study cancer-related goals of care in the ED. Because identifying cancer patients in the ED who may benefit from palliative or hospice care services can be challenging for clinicians, implementing clinical decision support tools, such as EHR facilitated assessments, could improve a cancer patient’s quality of life. A better understanding of specific palliative care or end-of-life needs would facilitate targeting such interventions. In addition, research is needed to identify interventions that minimize the need for ED care in those with advanced cancer or near the end of life, as well as better coordinate care after such visits occur.

## Designing and conducting oncologic emergency research

To support many of these research opportunities, workshop panelists and attendees stated that leveraging existing resources is key to generating knowledge related to oncologic urgent and emergency care. Several existing ED research networks were mentioned over the course of the workshop, including CONCERN, Geriatric Emergency care Applied Research (GEAR), Emergency Medicine Palliative Care Access (EMPallA), Pediatric Emergency Care Applied Research Network (PCARN), and the Multicenter Registry of potential COVID-19 in emERgency care (Project RECOVER). Additionally, oncology research networks, such as the NCI Community Oncology Research Program (NCORP), and existing cancer cohorts were noted as being potentially useful resources in conducting research related to oncologic complications. Although each resource has collected data with a specific focus — such as emergency medicine, dementia, palliative care, viral infections, and specific cancer types and treatment — they each represent opportunities to address identified evidence gaps.

As these existing resources highlight, workshop participants emphasized the role of persistent silos between specialties as hindering research and the delivery of optimal care. In particular, participants noted a separation between the emergency medicine and oncology departments (e.g., no dual appointments) that could be better bridged so that emergency medicine is recognized as an integral part of oncology care and research. Furthermore, the two disciplines tend to approach research differently (i.e., emergency medicine is more symptom-based, whereas oncology is organized by cancer type), and this distinction may have hindered collaborations. Participants also stated that many of the productive collaborations thus far have been at the individual investigator level rather than at a broader institutional level, and more effort to foster regional collaborations should be encouraged. Oncology and emergency medicine have a strong history of collaborating across disciplines where their clinical care and research interests intersect (e.g., oncology and cardiology for cancer-related cardiotoxicity and emergency medicine and neurology for stroke). These successful collaborative efforts should be replicated across oncology and emergency medicine at the investigator, institution, and professional organization level to help advance cancer-related urgent and emergency care research and expose junior clinicians and investigators to this emerging field.

Workshop participants emphasized the need for research efforts to understand and reduce disparities in care delivery experienced by underserved and underprivileged cancer patients who visit urgent care and the ED for unscheduled care. Cancer patients in rural areas with limited access to specialists experience higher rates of cancer-related mortality and suboptimal treatment outcomes compared to patients in non-rural areas [40]. To improve access to specialists, innovative interventions, such as enhanced telehealth in rural and community settings, could be evaluated, and any reduction in ED transfers to an academic medical center could be measured. Research efforts should optimally target translation of findings and interventions to a variety of care settings and populations to reduce disparities in care delivery among these patients.

Another aspect that is essential to future research efforts is to incorporate the patient perspective in all phases of research. The careful consideration of study design that reflects the needs of cancer patients is one important step that was noted. For example, including stakeholder-driven outcomes and comparators that are clinically justified and appropriate for the care setting and population is a way to better meet patient needs and preferences in the research setting [41, 42]. Understanding the unique perspectives of cancer patients and survivors who experience oncologic complications and designing future research studies that accurately reflect their needs are critical.

## Conclusion

This report highlights the progress made in cancer-related urgent and emergency care since the 2015 NCI and OECR workshop and identifies new research recommendations aimed at improving outcomes for those with unanticipated complications of cancer and its treatment. The research recommendations from workshop participants include the need to characterize the utilization of acute care services by cancer patients, predicting at-risk patients, care delivery models to minimize and support unplanned events, cancer prevention and screening efforts in the ED, management of cancer-related presentations, cancer-related goals of care conversations, and key considerations for designing and conducting research in this area. These research recommendations, coupled with the collaborative efforts of CONCERN and other scientific groups, have the potential to address the key evidence gaps and clinical care needs of cancer patients seeking urgent and emergency care.

## Data Availability

Not applicable.

## Abbreviations

CISNE: Clinical index of stable febrile neutropenia
CONCERN: Comprehensive Oncologic Emergencies Research Network
CORC: Coronavirus rule-out criteria
GEAR: Geriatric Emergency care Applied Research
ED: Emergency department
EHR: Electronic health record
EMPallA: Emergency Medicine Palliative Care Access
MASCC: Multinational Association for Supportive Cancer Care
NCI: National Cancer Institute
OECR: Office of Emergency Care Research
PCARN: Pediatric Emergency Care Applied Research Network
PERC: Pulmonary embolism rule-out criteria
Project RECOVER: Multicenter Registry of potential COVID-19 in emERgency care
